# The Early Clinical Outcomes Following Unrestricted Caliper Verified Kinematic Alignment Using a Medial Stabilized Design Total Knee Arthroplasty With a Cruciate Retaining Insert

**DOI:** 10.1016/j.artd.2023.101250

**Published:** 2023-10-22

**Authors:** Selin Munir, Leina Suzuki, Jorgen Hellman

**Affiliations:** a Medacta Australia, Lane Cove, New South Wales, Australia; b Orthopaedic Department, John Hunter Hospital, New Lambton Heights, New South Wales, Australia; c Orthopaedic Department, Lingard Private Hospital, Merewether, New South Wales, Australia

**Keywords:** Total knee arthroplasty, Cruciate-retaining, Medial stabilized, Kinematic alignment, Patient-reported outcome measures, Knee alignment

## Abstract

**Background:**

Although various total knee arthroplasty (TKA) implant designs are widely used, the ideal TKA design is yet to be agreed upon. Although the benefits of cruciate-retaining (CR) TKA and medial stabilized (MS) TKA have been reported in literature, the early clinical outcomes of an MS TKA with CR inserts have not been reported. This study aims to report on the patient-reported clinical and radiological outcomes of MS-TKA combined with a CR insert.

**Methods:**

A prospective single-surgeon series evaluated the clinical- and patient-reported outcomes of 115 patients implanted with GMK Sphere CR. Patient outcomes were assessed with the Oxford Knee Score, Knee Injury and Osteoarthritis Outcome Scores (KOOS), Forgotten Joint Score, and Visual Analogue Scale for Satisfaction. Radiological assessment for alignment along with active flexion and extension were also assessed.

**Results:**

Improvement in all scores was observed between the preoperative and 1-year follow-up timepoints, with statistical significance seen for Oxford Knee Score as well as KOOS Symptoms, Pain, Sport, quality of life, and activities of daily living subscales. The mean active flexion between the preoperative and both postoperative timepoints at 6 months and 1 year was also statistically significant (*P* = .021 and *P* = .001).

**Conclusions:**

MS-TKA with a CR insert can facilitate symptom relief and improve overall function of the knee after surgery. Both the patient and clinical outcomes were comparable to 1-year outcomes utilizing other MS-TKA designs and were superior to those at 1-year follow-up following implantation of CR-TKA. Most notability, the KOOS symptoms and sports score were higher for the MS-TKA with a CR insert than for an MS-TKA design.

## Introduction

Total knee arthroplasty (TKA) has revolutionized the treatment of patients with degenerative arthritis of the knee. Since its invention, the design of TKA has undergone significant revision and modification, with new implant designs and refinement of surgical techniques leading to improvements in clinical outcomes and survivorship rates [1,2]. However, currently there are approximately 19% of primary TKA patients who were not satisfied with their outcomes [3,4]. The difficulty in performing activities of daily life has significant influence on patient satisfaction and expectations. Many factors can affect the knee kinematics following TKA including the implant design. Although various TKA implant designs are widely used, the ideal TKA design is yet to be agreed upon or possibly yet to be developed.

The cruciate-retaining TKA (CR-TKA) centers around posterior cruciate ligament (PCL) retention with the assumption that preserving the native PCL is advantageous in aiding knee joint proprioception, reproducing physiologic knee biomechanics, and maintaining femoral rollback [[5], [6], [7], [8]]. Although some studies have reported superior proprioception and kinematics when the PCL is retained [[9], [10], [11], [12]], others have contrastingly suggested that sacrificing the PCL provides more natural kinematics with appropriate posterior femoral translation [5,13,14]. Although both older and contemporary posterior CR-TKA and substituting designs (PS-TKA) have been effective in providing pain relief and improved knee function, they have proven unsuccessful in replicating a predictable and physiological knee movement [15,16]. Irregular kinematics [17,18], abnormal patellar tracking [19], accelerated polyethylene wear [20], and poor range of motion (ROM) [21] have all been reported, compromising patient satisfaction.

The medial stabilized (MS-TKA) design was introduced in the early 1990s. Kinematic research demonstrated the medial compartment of the natural knee functioned like a ball-and-socket joint with the lateral femoral condyle translated in an anteroposterior direction and rotating around the medial compartment in flexion [[22], [23], [24]]. MS-TKA have been designed to have a conforming “ball-and-socket” medial femoral tibial articulation intended to confer anterior-posterior stability while the lateral femoral tibial articulation is sagittally unconstrained to allow the rollback observed in the natural human knee.

Although the benefits of CR-TKA and MS-TKA have been reported in literature, the use of a MS knee design with a CR insert to preserve the PCL has not been reported. The combination should provide inherent stability and improved kinematics from the CR insert while the MS-TKA provides AP stability through the ball-and-socket joint. Therefore, this study aims to report on the patient-reported outcome measures (PROMs) and clinical and radiological evaluation of a MS knee with a CR insert.

## Material and methods

The study was approved by the institutional research ethics committee. Patients who were scheduled for a TKA and met the inclusion criteria were invited to participate in the study and provide informed consent to be part of the study. Between September 2018 and November 2020, 177 MS-TKA (18 bilateral) were implanted with a GMK Sphere CR tibial insert (GMK-Sphere Knee; Medacta International, CSP Switzerland). All TKAs were performed by the senior author (J.H.) using the unrestricted caliper verified kinematic alignment (KA) TKA using manual instruments as per a previously described technique (Medacta International, www.medacta.com) [25,26]. The patella was resurfaced if there was significant preoperative patellofemoral irritability or a grade IV cartilage loss. The mean age of the study cohort was 69 years (45-90 years), where 49% were male. The cohort had an average BMI of 32.3 (21.0-54.0). Thirteen patients were unable to return to complete their 6-month clinical follow-up (1 bilateral), and a further 31 patients were unable to return to complete the 1-year clinical follow-up (1 bilateral, 1 left TKA of a staged bilateral, 1 right TKA of another staged bilateral). The final study cohort with complete data analysis consisted of 129 knees (115 patients), where 51% were male. The mean age was 69 years (45-90) with a mean BMI of 32.0 (22.0-49.0).

### Outcome measures

PROMs were the primary outcome measure for this study. Patients completed the Oxford Knee Score (OKS) and the Knee Injury and Osteoarthritis Outcome Score (KOOS) preoperatively and postoperatively at the 6-month and 1-year follow-up. The Forgotten Joint Score (FJS) and Visual Analogue Score (VAS) for Satisfaction were completed only at the postoperative timepoints. The secondary outcome was the clinical and radiological assessments. The clinical evaluation was assessed by measuring active knee flexion and knee extension both preoperatively and postoperatively at the 6-month follow-up timepoint. Radiological assessment for alignment was measured by the hip-knee-ankle angle (HKA), mechanical medial proximal tibial angle (mMPTA), and mechanical lateral distal femoral angle (mLDFA) via an anteroposterior long-leg weight-bearing radiograph.

To calculate the mMPTA, the joint line of the proximal tibia was drawn preoperatively across the flat aspect of the subchondral line of the 2 tibial plateau. Postoperatively, the proximal tibial line was drawn parallel to the undersurface of the tibial component. The medial angle formed by the intersection of the joint line and the tibial mechanical axis was the mMPTA. The varus alignment was classified as mMPTA less than 90°, and the valgus alignment as greater than 90° [27].

For the mLDFA, the preoperative joint line of the distal femur was drawn across the flat aspect of the subchondral line of the femoral condyles. The postoperative distal femoral line was drawn parallel to the undersurface of the femoral component. The lateral angle formed by the intersection of the joint line and the mechanical axis of the femur was the mLDFA. The valgus alignment was classified as mLDFA less than 90°, and the varus alignment as greater than 90°.

### Statistical analysis

Data were analyzed using SPSS, version 11 (IBM, Armonk, NY). Statistical significance was set at <0.05. The mean scores for the PROMs and clinical and radiological data were calculated for all timepoints and analyzed for statistical significance using the independent *t*-test.

## Results

### Patient-reported outcome measures

The patient outcomes were compared between the preoperative and postoperative timepoints and between both post-operative timepoints as shown in Table 1. The FJS and VAS Satisfaction were compared to the 2 postoperative timepoints. There was an improvement in all the scores between preoperative and 6-month follow-up. The mean difference in improvement at 6 months for the KOOS subscales was 34.1 Symptoms, 37.2 Pain, 35.6 activities of daily living (ADL), 41.4 Sport, and 48.0 quality of life (QOL). The mean difference in improvement for the OKS was 14.9. A statistically significant difference was seen for the KOOS Symptom and Sports score between the preoperative and 6-month follow-up timepoints (*P* = .0165 and *P* = .002, respectively). Similarly, an improvement in all the scores were observed between the preoperative and 1-year follow-up timepoints. The mean difference in improvement at 1 year for the KOOS subscales were 41.9 Symptoms, 45.8 Pain, 41.0 ADL, 52.3 Sport, and 56.9 QOL. The mean difference in improvement for the OKS was 20.4. There was a statistically significant difference between the preoperative and the 1-year postoperative follow-up, for the KOOS Symptoms (*P* = .001), Pain (*P* = .025), ADL (*P* = .005), Sport (*P* = .001), and QOL (*P* = .001), as well as for the OKS (*P* = .003). There was an improvement in the FJS (14.5) and the VAS Satisfaction (0.206) between the 6-month and 1-year follow-up timepoints; however, these were not statistically significant.

**Table 1:** PROMs result at preoperative, 6-month, and 1-year timepoints.

PROM	Subscale	Preoperative	Follow-up 6 mo	Follow-up 1 y	*P* value, preop. & 6 mo	*P* value, preop & 1 y	*P* value, 6 mo & 1 y
KOOS	Symptoms	43.7	77.8	85.6	.0165	.001	.006
	Pain	43.5	80.9	89.3	.803	.025	.015
	ADL	47.5	83.0	70.4	.517	.005	.028
	Sports	18.2	59.6	70.5	.002	.001	.337
	QOL	20.6	69.1	77.6	.083	.001	.562
OKS		20.8	37.6	41.2	.247	.003	.137
FJS		N/A	46.6	60.7	-	-	.552
VAS Satisfaction		N/A	86.1	89.3	-	-	.206

ADL, activities of daily living; N/A, not available; QOL, quality of life.

Statistical significance was set at *P* < .05.

### Clinical outcome

The active flexion and extension angles were captured and recorded in Table 2. There was an improvement in the active flexion angle of 7.6° (±11.4°) from the preoperative timepoint to 6 months and a statistically significant improvement from preoperative to 1-year postoperative timepoint of 12.1° (±9.8°) (*P* = .001). There was a statistically significant difference in the mean active extension between the preoperative and both postoperative timepoints (*P* = .001 and *P* = .001, respectively).

**Table 2:** Mean active flexion and extension angles at preoperative, 6-month, and 1-year postoperative timepoints.

Angle	Preoperative	6 Mo	1 Y	*P* value, preop. & 6 mo	*P* value, preop. & 1 y	*P* value, 6 mo & 1 y
Extension (°)	−5.6	−0.9	−0.7	.001	.001	.035
Flexion (°)	111.1	118.6	123.2	.021	.001	.178

Statistical significance was set to *P* < .05.

### Radiological outcome

The HKA angle, mMPTA, and mLDFA were captured preoperatively and at the 6-month postoperative timepoint (Table 3). The mean preoperative HKA angle was 4.0° varus (19.0° varus to 20.2° valgus). Fifty-seven percent of the cohort had a preoperative varus of greater than 4°, with 22.1% having a varus HKA of greater than 10°. Of the total, 16.3% had preoperative valgus of greater than 4°, with 3.8% having a valgus HKA of greater than 10°. The HKA was corrected to a mean of 0.8° varus (9.0° varus to 8.5° valgus). There was a statistically significant difference in the mean value between the preoperative and postoperative HKA angles (*P* = .001).

**Table 3:** Radiographic outcomes measured at preoperative and 6-month postoperative timepoints.

Radiographic outcomes	Preoperative	6 Mo	*P* value, preop. & 6 mo
HKA (°)	4.0 Varus	0.8 Varus	.001
mMPTA (°)	86.7	86.2	.438
mLDFA (°)	87.5	87.1	.929

HKA, hip-knee-ankle angle; mMPTA, mechanical medial proximal tibial angle; mLDFA, mechanical lateral distal femoral angle.

The mean preoperative mMPTA was 86.7° (80.7°-92.1°), where 90.6% of the patients had a varus alignment, 5.7% had a valgus alignment, and the remaining had a neutral alignment. The mean postoperative mMPTA was 86.2° (80.3°-91.4°). There was no statistically significant change in the mMPTA between preoperative and postoperative timepoints (*P* = .438). The mean preoperative and postoperative mLDFA were 87.5° (81.0°-96.3°) and 87.1° (80.5°-98.6°), respectively. There was no statistically significant change in the mLDFA between preoperative and postoperative timepoints (*P* = .929).

## Discussion

The search to optimize and mimic the native mechanics and restore optimum knee function has seen consistent advances and improvement in knee implant design. The advances in knee design have been led by the growing expectations of requiring optimized patient outcomes. The aim of this study was to examine the relative performance of an MS total knee design with a CR insert using an unrestricted caliper verified KA technique by assessing patient-reported outcomes and clinical outcomes. The design was developed to preserve the PCL combined with the advantages of the MS design. The literature has consistently presented a continuing debate about the superiority of CR-TKA over PS-TKA where there have been many studies comparing patient outcomes and obtaining joint-stability with functional improvement [28]. No notable difference has been observed in functional outcomes and ROM between CR and PS TKAs in most studies [28,29]. However, MS designs have shown to improve natural knee kinematics by addressing the paradoxical anterior femoral translation and providing stability throughout the arc of motion [30]. The GMK Sphere CR was introduced with the design rationale of utilizing an MS knee design with a CR insert that can accommodate the PCL.

The results of this study have shown that the implant performed well with improvements seen in both PROMS and clinical outcomes. Restoration of knee flexion is an important factor in determining the functional outcome following TKA; therefore, many studies report on the degree of flexion when discussing the outcomes of TKA. A study conducted by Devers et al. showed that an increase in knee flexion has a significant positive association of achievement of expectations, restoration of the normal knee, and functional improvements [31]. Meneghini et al. assessed the effect of the functional benefit of high flexion following TKA, where they compared the patient outcomes to the degree of flexion [32]. Meneghini et al. found no difference between patients that achieved a ROM greater than 115° compared to those with 125° in their Knee Society scores [32]. However, they did find that patients were more likely to demonstrate optimal stair function with increase in flexion angle. Similarly, Rowe et al. assessed the flexion angles recorded during daily functional activities, where they found that a minimum of 110° of flexion is required to complete activities of daily living such as walking, rising from a chair, and ascending/descending stairs [33].

This study has demonstrated that the MS design with a CR insert can improve ROM at 1-year follow-up, with a mean active flexion angle of 123.2° (±9.8°). Similarly, a fluoroscopy study conducted by Iwamoto et al. assessing the in vivo kinematics following a CR-TKA reported a mean ROM of 121° (±17.3°) [34]. The mean active flexion angle from this study was consistent with that in other reported early follow-up studies utilizing high-flexion CR-TKA [35,36]. However, when compared to a study conducted by Chaudhary et al. who assessed the early functional outcome following a standard CR-TKA, the mean active flexion was greater in the cohort from this study, with Chaudhary et al. finding a mean flexion of 105.9° (±13.0°) in the CR cohort [37]. A study by French et al. compared a CR-TKA to an MS-TKA and had shown a mean flexion angle of 114° and 115° at 1-year follow-up, respectively [38]. The current study demonstrated a greater active flexion at 1 year in comparison to both the MS-TKA and CR-TKA components. The higher mean flexion angle reported in this study may be contributed to the unique design features of the MS-TKA with a CR insert as the MS-TKA provides the ability to achieve the natural kinematics of the knee with the assistance of having the additional stability provided by retaining the cruciate. The specific design changes of the CR insert of having a reduced posterior congruency to accommodate femoral posterior rollback during flexion and introducing an anterior opening to accommodate the patellar tendon throughout the full ROM may have also contributed to the higher active flexion reported at the 1-year timepoint. Nedopil et al. [39] conducted a cadaveric study to compare the results of tibial rotation when the PCL was retained as well as excised using an unrestricted caliper verified KA. The study found that there was a loss of internal tibial rotation seen when the PCL was excised, which had caused extension loss and anterior liftoff in several knees. The authors also found that the loss of internal tibial rotation after PCL excision could not be corrected by changing tibial insert thickness or by reducing the posterior slope. Consequently, the authors concluded that PCL retention should be considered to match the native knee throughout flexion through the internal tibial rotation and coupled reduction in Q-angle. The results from the study by Nedopil et al. potentially provide additional support to the clinical findings of this study which has demonstrated higher ROM in comparison to other MS studies where the PCL is not routinely retained. The higher ROM can be possibly related to the retention of the PCL which Nedopil et al.’s study showed the excision of the PCL led to the loss of tibial rotation, causing extension loss. However, further research utilizing a large multi-author study is required as the clinical relevance of this is unknown.

The patient-reported outcomes demonstrated that the MS-TKA with a CR insert facilitates symptom relief and improves overall function of the knee joint after surgery. An improvement was seen as early as 6 months after the surgery, with a statistically significant improvement in both the KOOS subscales of Sport and Symptoms. In addition, the difference seen in KOOS scores at both 6 months and 1 year met the Minimal Clinically Important Difference (MCID) as established by Kuo et al. of 22.3 for Pain, 10.8 Symptoms, 22.1 ADL, 17.5 Sports, and 12.5 for QOL [40]. Similarly, the difference seen with the OKS at both 6-month and 1-year timepoints was significantly higher than the MCID of 5 [41]. The improvement in PROMs is important to achieve at 6 months after surgery as literature have reported that ongoing pain and persistent functional impairments are commonly at a higher risk of reporting diminished quality of life, difficulty performing activities of daily living, and poor physical and psychological health status [42]. If pain and function do not improve by 6 months, patients are more likely to be dissatisfied at 12 months and experience increased depressive symptoms [43,44].

Many studies have compared the clinical outcomes of the 3 different knee designs, where studies had found the superior performance of MS-TKA designs when reporting on CR-TKA, PS-TKA, and MS-TKA [38,[45], [46], [47]]. The improvements in PROMs from the preoperative stage to 1-year after surgery reported in the current study are consistent with other TKA studies which have also reported on improvement in patients’ pain, function, and quality of life resulting in the overall improvement in knee function [1,32,38,48,49]. Specifically, the KOOS scores for this study showed an improvement from preoperative timepoint to 1 year postoperatively for all subcategories; however, the quality-of-life subscore had the greatest improvement with a mean difference of 56.9 (23.6). When compared to the results of French et al., where the short-term functional and quality of life were compared between CR and MS-TKA, the mean improvement in patient symptoms, daily living, and quality of life were higher in the current study than with a traditional CR-TKA [38]. In addition, the mean improvement was greater for the present study for the KOOS subscales of Symptoms, Sports, and QOL than with the MS-TKA. The difference in the FJS at 6 months and 12 months did not meet the MCID of 16.6 established by Clement et al., with a difference of 14.1; however, it is worth noting that the difference was calculated by using the 6-month scores as the baseline rather than the preoperative timepoint used for the MCID calculation [50]. The overall FJS of 60.7 at 1 year is comparable to the FJS of other kinematically aligned MS implants reported in literature that ranges from 56.9 to 79.3 [1,45,51,52]. Similarly, in the study by Risitano et al. where patients were implanted with kinematically aligned MS implants, the OKS was similar to that in the present study, with the scores improving from 20.2 preoperatively to 41.3 at the final 1-year follow-up [52]. However, when compared to CR-TKA designs, the results of this study showed a greater mean difference in improvement in OKS score at early follow-up [53]. This finding can be a result of the improved knee kinematics to replicate the natural knee, which may allow the patient to feel more stable and natural with their joint during sporting activities. The MS-TKA design with a CR insert can lead to better kinematics and overall stability of the joint, which could be a potential factor to why the Sports and Symptoms subscale scores were higher than the MS-TKA results reported by French et al. [38].

The limitation of this study was that there was no comparator group to compare the outcomes of the GMK Sphere CR to a medial stabilized design undertaken by the senior surgeon. All TKA were also conducted by a single experienced surgeon with a limited follow-up of 1 year postoperatively. Due to the single-surgeon cohort, the patients are from a localized area; therefore, the study is not reflective of a generalized population. All clinical evaluations for the ROM and radiological evaluation were conducted by the senior author (J.H.) during consultations, which can be a potential source of reliability error with intraobserver and interobserver reproducibility. Forty-eight TKAs were lost to follow-up in this study, which may be a possible limitation, as 27% of TKAs were not reported on due to intraoperative decisions to use another insert type. As such, this may have introduced selection bias to the study cohort. Not all the patellae were resurfaced within the study cohort. An analysis comparing whether the resurfaced or non-resurfaced patellae would have an impact on the outcomes was not conducted. Therefore, this is another possible limitation to the study.

## Conclusions

In summary, we found the patient-reported outcomes demonstrated that the MS-TKA with a CR insert facilitates symptom relief and improves overall function of the knee joint after surgery. Both the patient and clinical outcomes were comparable to 1-year outcomes utilizing other MS-TKA designs and were superior to the 1-year follow-up outcomes following implantation of CR-TKA. Most notably, the KOOS Symptoms and Sports scores were higher for the MS-TKA with a CR insert than for an MS-TKA design. This agrees with the high mean flexion angle achieved at 1-year follow-up. These findings suggest that the MS design coupled with the CR insert is advantageous in facilitating improved functional outcomes and restoring the quality-of-life following knee surgery.

## Appendix A Supplementary data

Conflict of Interest Statement for Hellman

Conflict of Interest Statement for Suzuki

Conflict of Interest Statement for Munir
